# On the Necessity of the Minim Measure

**Published:** 1817-01

**Authors:** 


*For the London Medical and Physical Journal.*

### *On the Necessity of the Minim Measure;* by H. J. C. &c. &c.

IN your Journal for last month, there is a letter signed " Henry Ward," attaching blame to the College of Physicians for introducing the minim measure, and for endeavouring to abolish the custom of adding small doses of fluids by drops. A table is therein given, of which I will not dispute the accuracy, shewing the great difference that exists between the proportion of the drops of different medicines with each other and the measured minim. This very difference was the ground upon which was founded the necessity of introducing an alteration whereby the dose prescribed might be accurately known. If the minim be the sixtieth part of the fluidrachm of every tincture, spirit, wine, liquor, oil, &c. the practitioner, who cannot be supposed ignorant of the strength and virtues of what he may prescribe, well knows the quantity to be administered; but, if the drop may be either the 72d, or the 120th, or any intermediate part of the fluidrachm, what practitioner can know his dose? It is needless to add more; but let it be observed that Mr. Henry Ward has dropped his different medicines from a bottle of a particular size, and with a lip of a certain width. He well knows that the difference of the lip of the bottle makes a difference in the size of the drop; and, as different bottles are used by different apothecaries, the dose measured by drops must continually vary. My conclusion is, that the minim measure is not only an addition and improvement in the utensils of an apothecary's shop, but is absolutely essential in safe and correct practice.

